# Replicating Evidence-Based Practices with Flexibility for Perinatal Home Visiting by Paraprofessionals

**DOI:** 10.1007/s10995-017-2342-8

**Published:** 2017-07-28

**Authors:** Erin J. Rotheram-Fuller, Dallas Swendeman, Kimberly D. Becker, Eric Daleiden, Bruce Chorpita, Danielle M. Harris, Neil T. Mercer, Mary Jane Rotheram-Borus

**Affiliations:** 1 Division of Education, Leadership and Innovation, Arizona State University, Mail Code 1811, PO Box 871811, Tempe, AZ 85287 USA; 2 Department of Psychiatry and Biobehavioral Sciences, Semel Institute, University of California at Los Angeles, 10920 Wilshire Blvd., Suite 350, Los Angeles, CA 90024 USA; 3 Department of Psychology, University of South Carolina, 1512 Pendleton Street, Columbia, SC 29208 USA; 4 Practicewise, LLC, 340 Lee Ave, Satellite Beach, FL 32937 USA; 5 Department of Psychology, University of California at Los Angeles, 1285 Franz Hall, Los Angeles, CA 90095 USA

**Keywords:** Intervention fidelity, Peer social support, Maternal depression, Maternal body mass index

## Abstract

*Introduction* Strategies are needed to improve the efficacy of paraprofessional home visitors for pregnant women in the United States. This study evaluates the maternal and child outcomes when evidence-based practices (EBP) are replicated with flexibility, rather than fidelity to a manualized intervention. *Methods* Pregnant mothers (N = 203) in five clinics were recruited in the waiting rooms and randomized to standard clinic care as the control condition (n = 104) or standard care plus home visiting (n = 99). Home visitors (n = 9) were selected, trained in foundational skills common to EBP and four problem domains (weight control, breastfeeding, daily habits, and depression). Independent interviewers assessed targeted outcomes at birth (82%) and 6 months later (83%). *Results*: Home visitors, called Mentor Mothers [MM], made an average of 14.9 home visits or telephone contacts (SD = 9; total contacts = 1491) addressing maternal daily habits, breastfeeding, and depression. Intervention and control mothers were similar in weight, Body Mass Index (BMI), depression and social support at baseline and 6 months later. The percentage of low birth weight babies was similar; intervention infants’ growth (weight/height Z score) tended to be significantly better compared to the control condition. *Discussion*: There are many explanations for the failure to find significant benefits: insufficient statistical power; the benefits of repeated assessments by warm, supportive peers to improve outcomes; or the failure of EBP and the need to maintain replication with fidelity. All study mothers had better outcomes than documented among comparable published samples of low-income, Latina and Korean–American mothers in Los Angeles, CA. ClinicalTrials.gov registration NCT01687634.

## Significance

Strategies are needed to improve the efficacy of paraprofessional home visitors to support pregnant women to achieve health outcomes in the United States. Even in efficacious programs, there is often high turn-over of paraprofessional staff, many mothers reject home visitors, and the implementation quality has been questioned when home visitors have minimal training. This study experiments with the existing scientific norms of “replicating with fidelity” evidence-based intervention manuals. Although there were not robust outcomes across groups, this provides testing of one alternative model that had real benefits in uptake and implementation.

## Introduction

The efficacy of nurse home visiting among low-income, first-time mothers has been previously demonstrated (Olds et al. 2014). In response, researchers and public health providers have sought to achieve similar results at lower cost by task shifting from nurses to paraprofessionals (Sweet and Appelbaum 2004). A review of 60 studies evaluating these efforts (Peacock et al. 2013) found that there are substantial problems when implementing home visiting with paraprofessionals. There have been at least eight different programs which have been seen as having positive results (Administration for Children and Families Office of Planning Research and Evaluation 2015). Yet, even in these efficacious programs, there is often high turn-over of paraprofessional staff; many mothers reject the home visitors and the implementation quality has been questioned as training typically lasts only about a week (Witkin 2013). The outcomes are not as efficacious with paraprofessional home visitors (Olds et al. 2014). Yet, there are at least 200 counties in the United States that are implementing home visiting with paraprofessionals (http://www.acf.hhs.gov).

This study experimented with implementation strategies that could increase the impact of paraprofessional home visitors and their programs. The current standard for diffusing evidence-based (EB) interventions is to replicate manuals with fidelity (Flay et al. 2005). When EB interventions are diffused, however, this standard is not typically followed and manuals are not replicated with fidelity. This study examines an alternative strategy for selecting, training, and monitoring home visitors that is based on replicating with flexibility EBP using coaching, not a manualized intervention. Rather than seeking fidelity to a sequenced set of activities and scripts, home visitors are taught the practices and skills common across evidence-based interventions for children and families. In addition, the home visitors are taught to focus these skills on securing day-to-day implementation of healthy habits by pregnant and new mothers that will lead to the desired outcomes.

Current evidence-based interventions have much more in common with each other than is unique, especially programs that address the same outcomes (Rotheram-Borus et al. 2014). Almost all EB interventions are based on a cognitive–behavioral theory of change (Meichenbaum 1977). Almost all rely on the creation of a supportive relationship between a change-agent and a participant (Bergin and Garfield 1994) to motivate engagement in the intervention. Increasingly, reviews of preventive interventions and psychotherapeutic interventions for children and families find many common skills and practices across EB interventions. For example, in psychotherapy literature, Chorpita and Daleiden (2009) have found that 14 common practices are shared by 80% of the EBP for childhood disorders. Rotheram-Borus et al. (2014) found that preventive adolescent HIV interventions have highly active, goal-focused leaders who reward youth frequently within each meeting. These reviews suggest that rather than replicating a manual with fidelity, psychotherapists would benefit from using the skills employed by EB interventions for the same problem behavior. When training therapists in this way, the results are about 2.5 times more efficacious than in a typical evidence-based intervention for the same disorder (Chorpita et al. 2013).

This team extended the approach of Chorpita and Daleiden (2009) with professional therapists to paraprofessionals. Paraprofessionals typically need far greater training than professionals and, even then, it is unclear whether similar results can be achieved by paraprofessionals. In our work in South Africa with community health workers conducting home visits (le Roux et al. 2013; Rotheram-Borus et al. 2014; Tomlinson et al. 2016), we found significant benefits of perinatal home visiting over 3 years. Home visitors were selected who were positive role models in their communities. Each was trained initially and on an ongoing basis with supervisors supplied with data from every home visit; and the home visitors were monitored via mobile phones at the end of each visit, including with a time and location stamp (Rotheram-Borus et al. 2011). Home visitors were taught a skills-based coaching model. Cognitive–behavioral theory was translated into: mothers change slowly, over time, with small steps, in relationships, with opportunities, and rewards. Home visitors translated this theory into coaching with the skills common across evidence-based interventions (Chorpita and Daleiden 2009). At every visit, the home visitors reported which skills were utilized in coaching the women how to achieve the targeted outcomes of maintaining a healthy weight, breastfeeding their baby, and engaging in pleasant events (to buffer against depression). By monitoring the skills used in each encounter and the topics discussed, the supervisors knew what topics to review in supervision. Finally, in order to provide a demonstration model, all home visitors were shown the evidence-based home visiting intervention, Partners for a Healthy Baby. Partner’s for a Healthy Baby is an evidence-informed home visiting intervention program with a manual designed to address issues surrounding child development over the first 36 months of life. The program is used nationally and internationally by over 3700 parenting and family support programs. We did not ask the home visitors to replicate any part of this manual; home visitors read and practiced scripts from the curriculum as a model of ways other people coached pregnant and new mothers. Home visitors were encouraged to use their own words, their knowledge of their culture and their community to achieve similar goals with the mothers in Los Angeles. Given these selection, training, and monitoring procedures, the impact of delivering perinatal home visits was evaluated in a pilot study over 6 months.

The key issues to be addressed by perinatal home visitors vary based on the community risk factors. Nationally and in Los Angeles, the four goals for pregnant and new mothers are: (1) reducing maternal obesity and helping women return to pre-pregnancy weight, (2) prolonging breastfeeding, (3) reducing depression, and (4) increasing infants’ health. About half of African American and Latino women are overweight when becoming pregnant (Keppel and Taffel 1993), and are likely to retain about 14 lbs. at 6 months post-birth (Institute of Medicine and National Research Council Committee to Reexamine IOM Pregnancy Weight Guidelines 2009). To reduce the women’s risk of cardiovascular disease and diabetes, these patterns must change (Institute of Medicine and National Research Council Committee to Reexamine IOM Pregnancy Weight Guidelines 2009).

Post-birth, breastfeeding is a critical advantage for lifelong management of obesity for both mother and child (Armstrong and Reilly 2002). Pregnant women typically adopt more sedentary routines and are unaware of the consequences of obesity (Keppel and Taffel 1993). Breastfeeding helps women reduce weight gained during pregnancy. Yet, there is a far more important reason to breastfeed. Maternal breastmilk has nutrients and immunities that protect the baby during the first few years of life (Horta et al. 2007). To maintain breastfeeding, many barriers must be overcome. For example, Latina mothers in our sample reported that their husbands and partners did not find them sexually attractive while breastfeeding. There is also a belief that breastmilk is insufficient for babies after 2 months of age, and 68% of Latina women in Los Angeles use more than one feeding method while still in-hospital after childbirth (California Department of Public Health 2010). We tried to directly address these maternal barriers and encourage each woman to breastfeed for the child’s well-being and to facilitate her own weight loss. Healthy eating, routine exercising, refraining from smoking and drinking alcohol were considered critical for reducing maternal obesity and improving children’s growth and well-being.

About 10% of women suffer from perinatal depression (Ertel et al. 2011). In Los Angeles County, screening for perinatal depression is not widespread or standardized (Spatzier et al. 2009). The Los Angeles Mommy and Baby Project demonstrated that up to 52% of new mothers reported depressive symptoms (LA Best Babies Network 2009). In this study, African American and Latina women were more likely to self-report feeling depressed, as well as younger and poorer women (LA Best Babies Network 2009). Chronic disease is an additional risk factor for maternal depression; among low-income mothers, pre-pregnancy or gestational diabetes has been repeatedly associated with perinatal depression (LA Best Babies Network 2009). Low birthweight babies comprise 7% of all live births in Los Angeles, and are most common among African American women and women without antenatal care (California Department of Public Health 2013). Antenatal care has been proven beneficial to increasing infant health, however low-income families are less likely to have access to this care (Finlayson and Downe 2013). Given the rates of depression among Latino mothers and children living in low-income families in Los Angeles, these were two issues monitored in this study. Social support often buffers depression (Ammerman et al. 2015). Both Latino and Korean cultures are more group-oriented than individualistic, increasing the importance of social support (Hyun 2001; Sabogal et al. 1987). Thus, home visitors were encouraged to help women maintain their social contacts during pregnancy and the stressful time post-birth.

We examined the efficacy of an intervention approach grounded in the therapeutic practices that are common across EBP and that include selection of paraprofessionals who are positive role models, provision of data-informed supervision, and real time monitoring over time. Using these practices to address the content areas reviewed of an established curriculum, we established obesity, breastfeeding, depressed mood, and infant growth as the primary targets in the first 6 months post-birth.

## Methods

### Sites

The Pico-Union area of Los Angeles (ZIP codes 90006, 90007, 90015) is more than two-thirds Latino, with a sizable Asian population (16%) (U.S. Bureau of the Census 2010). Pico-Union is often the arrival community for immigrants, especially from Mexico, El Salvador, and Korea (LA Times 2000). Pregnant women in these neighborhoods typically receive antenatal care at local clinics, Federally Qualified Health Care Clinics (FQHC) or FQHC look-alikes. We recruited 202 pregnant women from the waiting rooms at five local clinics over a period of 9 months. Women were typically fluent in Spanish and/or Korean, as their first language. This study was approved and monitored by the University of California, Los Angeles Institutional Review Board, and all ethical guidelines were followed.

### Assessments

Nine local parents from a K-12th grade school, who were fluent in Spanish or Korean, served as interviewers for the project. Pregnant women were met in the waiting room and scheduled an assessment interview at the woman’s convenience. The interview could take place at home, at a local school, or a nearby church. Women were interviewed using a pre-programmed mobile phone which prompted queries about the following issues at a baseline assessment, within 2 weeks of birth and 6 months later.

### Sociodemographic Characteristics

Age, education, marital status, household composition, income, and previous children were reported.

### Outcomes

Maternal *Body Mass Index (BMI*) was calculated for each assessment point. Height was recorded at the baseline and weight was self-reported for prior to pregnancy. Women were weighed at 2 weeks and 6 months post-birth.

Breastfeeding was reported at each assessment; interviewers also looked for signs of breastfeeding—nursing bras, baby formula—and asked other family members about food practices. Additionally, the Edinburg Perinatal Depression Inventory (EPDS; Cox et al. 1987) and General Health Questionnaire (GHQ-12; Goldberg and Williams 1988) were delivered. Social support was measured by counting the women’s number of close relatives and friends, and averaging the frequency of contact with their own mother, father and partner; of receiving practical support; and, participating in recreational events.

Child’s growth was measured repeatedly, with the length and weight and head circumference recorded; each interviewer was trained in how to weigh and measure the length of infants prior to beginning data collection. The measurements were converted into weight-for-age Z-scores, height-for-age Z-scores, and weight-for-height-for-age Z-scores (WFHA) based on the scales of the World Health Organization (World Health Organization & United Nations and Fund 2009). Children who were more than 2 SD below the WHO norms for weight were considered undernourished and more than 2 SD below in height were considered stunted; being above 2SD were over-nourished or tall.

### Home Visiting Interventions

Mentor Mothers (MM) were local parents who were perceived to be socially skilled and positive role models in their community. MM were trained in foundational skills (called practice elements) common across EBP (Chorpita and Daleiden 2009) for 1 week. MM then received a month of training, which included educational information about how to achieve each targeted outcome (e.g., tips about breastfeeding). For each issue, the MM practiced how to:

- frame each targeted behavior (e.g., breastfeeding protects your child early and has lifelong benefits);
- provide information that had to be applied to the participant’s life (breastfeeding will hurt in the beginning, but you can prepare your breasts and know that the tenderness will stop in about a week);
- build the skills and routines that would help the mother consistently implement a new behavior;
- provide social support for new routines; and
- eliminate environmental barriers to achieving the targeted outcome behavior.

Finally, training included review of the manual from the Partners for a Healthy Baby curriculum. MM were not asked to implement any part of this program, but to use it as a demonstration model of what other home visitors had done. MM were also trained in basic skills to conduct home visiting: how to enter a household, bond with pregnant and new mothers, deal with crises situations, and how to use a mobile phone reporting system. For each visit, MM reported whether a home visit or telephone call occurred, the data and global positioning system (GPS) location where this interview was conducted, the content area covered in the visit/call, and the types of practice elements employed. The MM’s contacts were also time and location stamped automatically by the telephone.

Each meeting was planned in a set sequence: identify strengths or successes from the previous week; identify a new topic for the day; identify how that topic was challenging for the mother and her child; practice and problem solve how to address this challenge; review the progress of the day; and set a new goal for the next week. These procedures were common across not only this intervention, but many preventive intervention sessions.

Overall MM conducted 1431 home visits with an average of 14.9 contacts per mother in the home visiting condition with contacts lasting about 31 min each. The topics discussed, the practice elements used and the relationship between these process measures and outcomes have been reported previously (Rotheram-Fuller et al. 2017).

### Analysis

Random effects linear regression models (Gibbons et al. 2010) were used to compare continuous outcomes for depression, social support, and WFHA Z-scores between intervention conditions over time. Models included covariates for intervention, time, and a two-way interaction between intervention and time. Intervention effects were indicated by statistically significant interactions. Random effects were included for each mother to allow the intercept for each mother’s outcome trajectory to differ from the overall intercept estimated across mothers, i.e. random intercepts (RI) were estimated for each mother. The RI adjusted standard errors of the regression coefficients to account for correlations between repeated outcome observations and provide valid statistical inference. The number of assessments varied for different outcomes, because Mentor Mothers weighed the mothers at each visit. Therefore, models included different numbers of outcome observations over time based on the number of outcome observations available.

## Results

### Pregnant Women

Table 1 summarizes the pre-pregnancy and baseline reports of pregnant women. The average age of the pregnant women at recruitment was 27.9 years (SD = 6.1), and 81.5% were Spanish speakers. Significantly more intervention mothers planned their pregnancies compared to control mothers. Only 19.8% of the pregnant women had graduated from high school. Almost one quarter (23%) were single while the rest were married or had a live-in partner. Only 48% of families had a regular source of income and 51% were on public assistance. More than 90% had less than $2000 a month income, with 53% having less than $1000 a month income. About half the babies were planned and 23% of mothers went hungry at least 1 day in the last week. Only 33% of mothers had a BMI of 25 or less (in the normal range) prior to pregnancy, based on self-report. About one-third were obese prior to pregnancy, and another third were overweight. About 30% of mothers-to-be were depressed. As much as 24% of women used alcohol while pregnant. Roughly 12% of mothers had a previous low birth weight baby (<2500 g). Almost all women had been tested for HIV (94%). Mothers in the control condition were twice as likely to have TB compared to mothers in the intervention condition (p < .05), but overall rates of TB were low. Most mothers in Pico-Union area had family meals in the last week. Data that suggests Hispanic and Asian–American families report sharing meals at a higher rate compared to other ethnic groups (Larson et al. 2006).

**Table 1:** Sample characteristics at the baseline interview grouped by the intervention (n = 99) and control (n = 104) conditions

Descriptive variable	Intervention (n = 99)		Control (n = 104)		Total (N = 203)	
	M (n)	SD (%)	M (n)	SD/%	M (n)	SD (%)
M age (SD) in years	28.5	5.6	27.8	6.4	28.1	6.0
M weeks pregnant (SD)	24.4	9.4	25.9	9.3	25.2	9.3
Baby planned	50	50.5%	38	36.5%	88	43.3%*
Mother’s language at home						
English	15	15.2%	12	11.5%	27	13.3%
Spanish	80	80.8%	87	83.7%	167	82.3%
Korean or other	4	4%	5	4.8%	9	4.4%
Mother’s education						
≤ Some high school	61	61.6%	67	64.4%	128	63.1%
≥ High school graduate/GED	38	38.4%	37	35.6%	75	36.9%
Marital status						
Single	21	21.2%	22	21.2%	43	21.2%
Married/living together	78	78.8%	82	78.8%	160	78.8%
Receiving social welfare	18	18.2%	16	15.4%	34	16.7%
M household members (SD)	4.2	1.7	4.4	1.7	4.3	1.7
Current partner	82	82.8%	91	87.5%	173	85.2%
Father living in household	72	72.7%	82	80.4%	154	76.6%
Unemployed	78	78.8%	80	76.9%	158	77.8%
Regular household income	50	50.5%	55	52.9%	105	51.7%
Household public assistance	52	52.5%	56	53.8%	108	53.2%
Household monthly income (USD)						
<$1000	43	43.5%	49	47.1%	92	45.4%
$1001–2000	42	42.4%	43	41.3%	85	41.9%
$2001–$5000	10	10.1%	9	8.7%	19	9.4%
Don’t know	4	4%	3	2.9%	7	3.4%
Maternal health in pregnancy						
M Height (SD)	61.0	3.8	61.0	3.3	61.0	3.5
M Weight (SD)	160.7	37.2	155.3	32.7	158.0	35.0
M BMI (SD)	30.4	6.1	29.2	5.3	29.8	5.7
% Underweight/normal	33	33.3%	38	36.5%	71	35%
% Overweight	34	34.3%	40	38.5%	74	36.5%
% Obese	32	32.3%	26	25%	58	28.6%
Chronic illness						
Diabetes, hypertension, asthma, and/or positive TB result	22	24.1%	41	43.7%	63	34.2%
Depression						
M EPDS score (SD)	6.9	5.3	7.4	4.9	7.1	5.1
EPDS (score > 13; caseness)	13	13.1%	12	11.5%	25	12.3%
M HQoL, range 0–36 (SD)	9.8	6.2	10.1	4.9	10	5.6
GHQ (score > 13)	23	23.2%	20	19.2%	43	21.2%
GHQ (score > 9; caseness)	40	40.4%	43	41.4%	83	40.9%
Alcohol use prior to pregnancy	21	21.2%	22	21.2%	42	21.2%
Frequency of social support	26.9	48.7	30.4	89.3	28.7	72.2

*M* Mean

*p < .05

### Intervention Delivery

Using mobile phones, MMs addressed daily habits (63%), breastfeeding (54%), parenting and child development (29%) and medical adherence (21%) as the content domains regularly reviewed. During phone calls, the practice elements used most often include attending, goal setting and relaxation (46%), praise and rewards (43%), monitoring (37%) and modeling (21%).

### Maternal and Child Outcomes

Table 2 summarizes the outcomes over 6 months post-birth. Women’s BMI was similar across conditions at pre-pregnancy, at the baseline interview, and 6 months later. Intervention mothers did not gain less weight during pregnancy nor lose more weight post-birth than mothers in the control condition. Women retained, on average, 2 lbs from their pre-pregnancy weight at 6 months post-birth.

**Table 2:** Outcomes over the first 6 months of life for mothers and children in the intervention (n = 99) and the control (n = 104) conditions

Mothers	Intervention (n = 99)				Control (n = 104)			
	Pregnancy		6 Months		Pregnancy		6 Months	
	M (n)	SD (%)	M (n)	SD (%)	M (n)	SD (%)	M (n)	SD (%)
Maternal weight^a^								
M Weight (SD)	149.6	42.3	155.7	40.5	143.3	31	149.5	33.1
M BMI (SD)	28.2	6.8	29.4	6.8	27	5.2	28.2	5.8
Maternal mental health								
EPDS (SD)	6.9	5.3	3.9	4.1	7.4	4.9	4.2	4.9
EPDS (score > 13)	13	13.1%	1	1.3%	12	11.5%	5	5.9%
M HQoL, range 0–36 (SD)	9.8	6.2	7.7	4.6	10.1	4.9	8.5	5.3
Any Alcohol Use^b^	21	21.2%	16	20%	22	21.2%	16	18.82%
Frequency of Social Support	18	18.2	11	13.8	16	15.4	10	11.8

Children	Intervention (n = 99)				Control (n = 104)			
	Baseline		6 Months		Baseline		6 Months	
	M (n)	SD (%)	M (n)	SD (%)	M (n)	SD (%)	M (n)	SD (%)
% Low birth weight			6	7.1%			8	8.42%
Growth								
Height-for-Age Z-Score	−0.2	2.0	0.8	4.5	0.0	2.1	0.8	1.9
Weight-for-Age Z-Score	0.2	1.2	0.3	1.0	0.1	1.2	0.2	1.2
Weight-for-Height-by-Age Z-Score	0.1	1.8	0.2	1.3	−0.2	2.0	−0.1	1.5
Duration of breastfeeding								
<1 Week			16	20.78%			18	22.5%
1 Week–2 Months			23	29.87%			29	36.25%
>2 Months–6 Months			46	59.74%			53	66.25%
6 Months			31	40.26%			27	33.75%

*M* Mean

*p < .05

^a^Pregnancy weights are self-reported

^b^Any alcohol use before pregnancy

The duration of breastfeeding was similar across conditions. Babies tended to be breastfed longer than the median number of months in the intervention and the control compared to published reports of duration of breastfeeding in Los Angeles County (Los Angeles County Department of Public 2013). Ten percent more women (60 vs. 50%) in the intervention condition breastfed their children for more than 3 months.

The mean depression and quality of life of women was similar post-birth and 6 months later, as was the percentage of women who met a criteria indicating likely depression (i.e. >13 on the EPDS and the HQoL > 9). Social support was also similar across conditions at baseline and 6 months.

The rate of low birth weight infants (<2500 g) was similar across condition (8.4 control; 7.1 MM). Infants in the MM condition tended (p < .07) to have better WFHA Z-scores; however, infant growth was similar on other measures of height-for-age Z-scores and weight-for-age Z-scores.

## Discussion

Domestically, many paraprofessional home-visiting programs addressing mothers’ and children’s health have had highly variable results (Filene et al. 2013). Six review articles evaluating home visiting over the last 10 years have also found variations for multiple health-related tasks including breastfeeding (Morrow et al. 1999) and mother-infant attachment (Cooper et al. 2009). This study continues those mixed results. Overall, there are no significant differences between the intervention and control conditions at any of the time points. However, with a sample size of 203, there is less than 30% power to detect a significant effect. Had the observed effect size been accurate, it would have required a sample size of 500 women per condition to achieve a significant difference on BMI, the duration of breastfeeding, or depression. Because weight was self-reported prior to pregnancy, mothers may have over-reported their pre-pregnancy weight, being biased towards their current weight. Biased reporting would decrease in the likelihood of finding effects. Many control groups in randomized controlled trials improve 30% (Rotheram-Borus et al. 2014). The interviewers were socially skilled local women from similar backgrounds to the sample. There may have been an intervention effect of having a supportive peer repeatedly inquiring about your health status, especially your weight and mood. Finally, our measurement tools may be insensitive and not reflect differences between the conditions.

This study was intended as a pilot study and the results demonstrate feasibility of training paraprofessionals with practice elements, content areas, and the ability to bond with pregnant and new mothers. These three goals were achieved with no MM being rejected, and the mobile phone data documenting that MM addressed the targeted outcomes for a mean of 14.9 sessions with each targeted mother. However, there is little demonstration of an impact on outcomes. Future studies can benefit from the addition of new measures such as testing cortisol levels, measuring overall well-being, and more closely examining MM who had significantly positive results. By examining the qualities of successful mentors, the intervention tools can be further improved.

The trends in positive outcomes shift in the desired direction. Even though there tends to be some gain in WFHA Z-scores for infants, the children in this study children are not under-nourished. These children are doing well at 6 months.

However, these data are very different from the most recent data on weight gain among pregnant women in Los Angeles County (Los Angeles County Department of Public Health 2013). In Los Angeles County, nearly half (41%) of mothers gain more than Institute of Medicine’s pregnancy weight gain recommendations. African American, White and Latina women are most likely to gain more weight than recommended. African American and Latina women are more likely to be overweight or obese prior to pregnancy, and more than half of overweight and obese women gain more weight than recommended (Los Angeles County Department of Public Health 2013). Childhood obesity is an epidemic in Los Angeles County; more than 20% of 5th, 7th and 9th graders are considered obese (Los Angeles County Department of Public Health 2013). In the Metro Service Planning Area (SPA) that encompasses the Pico-Union neighborhood, this figure rises to more than a quarter among preschool children (Los Angeles County Department of Public Health 2013). Overall, all women and children in this study were doing better on the targeted outcomes, compared to other published reports of mothers and children in Los Angeles.

Compared with nurse home visitors, peers are potentially: (1) less expensive than nurses and a more easily accessed workforce; (2) able to network neighborhood mothers with each other, creating an ongoing source of social support over time that may serve emotional, social, and instrumental functions; and (3) likely to be better problem solving experts, as role models, than professionals in the local neighborhood (Olds 2014). Yet, if these MM cannot produce evidence-based outcomes, the utilization of paraprofessionals benefits few.

Globally, the trend towards task-shifting has become a major initiative, with calls for every country to allocate resources to paraprofessional home visitors to improve health and child outcomes (Sachs and Sachs 2015). Yet, the results of this U.S.-based trial point to the importance of focusing on the implementation of home visiting by paraprofessionals and questions our current investments in this approach. This study does provide one example of how to experiment with our existing norms of “replication with fidelity.” This article presents one potential model as an alternative way to utilize existing EBP, but use the information from past work in a novel fashion. Clearly, based on our failure to achieve robust outcomes, this is not the only adaptation model to consider. However, we have presented one alternative model that had benefits in uptake and implementation.
